# Solving the degradation problem of continuous ceftazidime infusions—the Cooltaz study

**DOI:** 10.1128/aac.01385-25

**Published:** 2026-05-29

**Authors:** Sonia L. La Vita, Beverley Liu, Penelope A. Bryant

**Affiliations:** 1 Murdoch Children’s Research Institute, Melbourne, Victoria, Australia; 2 Department of Paediatrics, The University of Melbourne 2281, Parkville, Australia; 3 Hospital-In-The-Home Department, The Royal Children’s Hospital, Melbourne, Australia; 4 Infectious Diseases Department, The Royal Children’s Hospital, Melbourne, Australia; University Children's Hospital Münster, Münster, Germany

**Keywords:** antibiotics, ceftazidime, pyridine, outpatient parenteral antimicrobial therapy, hospital-based home care services, hospital at home, hospital in the home

## Abstract

Ceftazidime is an effective antipseudomonal antibiotic, used commonly in cystic fibrosis. Continuous 24-hour intravenous infusion allows children to receive daily treatment at home. However, the instability of 24-h ceftazidime infusions and production of toxic pyridine has led to recommendations not to use them. We aimed to determine a suitable target temperature to limit degradation of ceftazidime in 24-h infusions and assess effectiveness of simple temperature control. For *in vitro* experiments, infusers with 6g ceftazidime in 0.9% saline were incubated at 4ºC, 25ºC and 33ºC and sampled over 24h to measure ceftazidime and pyridine concentrations using high-performance liquid chromatography. For carried infuser experiments, to simulate 24-h home infusions, children carried two insulated carry bags containing ceftazidime 2g, 6g or 12g infusers with zero, 1 or 2 ice-packs for 24h. Samples were analysed for ceftazidime and pyridine concentrations. Results for *in vitro* experiments showed that after 24h, ceftazidime remained stable (>90% original concentration) at 4ºC and 25ºC but not 33ºC (decreased to 84%). Pyridine increased over 24h at 4ºC, 25ºC and 33ºC by 50%, 550% and 1,040% respectively. For carried infuser experiments, at a controlled temperature of 17-25ºC, ceftazidime concentration remained >90% for all infusers except 12g with no ice-pack. Pyridine production in infusers with one or two ice-packs were reduced by 44-57% compared to no ice-pack. In conclusion, adding an ice-pack to a ceftazidime infuser in an insulated carry bag halves the pyridine produced. This indicates that a temperature-controlled daily 24-h continuous intravenous infusion of ceftazidime can replace the recommended twice daily 12-h infusions, allowing patients to receive ceftazidime treatment at home.

## INTRODUCTION

Children with cystic fibrosis need frequent antibiotic treatment for infective respiratory exacerbations, without which their lung function inexorably declines. Intravenous ceftazidime is a mainstay of treatment for these exacerbations (1, 2). This is because, despite being a third-generation cephalosporin, it is one of the narrower-spectrum options that has activity against *Pseudomonas aeruginosa*, a major pathogen determining outcomes in cystic fibrosis. Continuous intravenous infusion of ceftazidime over 24 h has improved bactericidal efficacy compared to intermittent dosing (3–6). When delivered via portable infusers, this allows children to receive outpatient parenteral antibiotic therapy (OPAT) at home via hospital-in-the-home (HITH) programs (7–9). HITH models are cost-effective and reduce the chances of hospital-associated adverse events, but critically for children with cystic fibrosis, the ability to be treated at home helps minimize the psychological impact of chronic disease (10–12).

Laboratory-based studies have highlighted the degradation of ceftazidime when infused over 24 h at body temperature, below the recommended minimum concentration of 90% of the original concentration (13–15). While lower temperatures reduce degradation, drug delivery is impaired due to increased viscosity and impact on flow regulator function. As ceftazidime degrades, a toxic by-product pyridine is produced (15–17). Pyridine ingested in large quantities can cause vomiting, neurological symptoms, and even death. However, it is also present environmentally and in many foods, so some exposure is considered universal. Countries including Australia follow the European Medicines Agency (EMA) and US Pharmacopeia (USP) guidelines, which have set an upper limit of pyridine concentration in drug infusions at 0.2 mg/mL and environmental pyridine exposure at 2 mg/day (18).

Although there are no reported cases of adverse effects caused by pyridine from 24-h ceftazidime infusions (19), a study showing increased pyridine production at higher temperatures and longer infusion time led to recommendations to stop using 24-h infusions (15, 16). Globally, clinicians have changed to twice-daily 12-h infusions (increasing the cost and reducing the ability of HITH services to provide this treatment), used broader antipseudomonal antibiotics (contributing to increased development of antimicrobial resistance), or simply elected to keep these patients in hospital and treat them with intermittent dosing. None of these is an ideal solution.

The best solution would be to find a way to stabilize ceftazidime so that pyridine production is reduced. Because ceftazidime is more stable at lower temperatures, we aimed (i) to determine a suitable target temperature of ceftazidime infusion *in vitro* and (ii) to assess whether this could be replicated in the practical setting using carried 24-h infusers.

## MATERIALS AND METHODS

### Study design

There were two stages to this study: *in vitro* and *in situ*. In the *in vitro* stage, ceftazidime infusers were incubated for 24 h in laboratory-controlled conditions, and ceftazidime and pyridine concentrations were measured. Based on these findings, the *in situ* stage simulated the HITH setting, with carried ceftazidime infusers, and ceftazidime and pyridine concentrations were again measured.

### Materials

Ceftazidime powder, as pentahydrate (2 gram [g] vials, Fresenius Kabi, Australia), was reconstituted with Milli-Q water from a Millipore purification system (Sigma-Aldrich Inc., Australia). Sterile 0.9% sodium chloride (Baxter Healthcare Pty Ltd., Melbourne, Australia) was added to make a total volume of 240 mL in Infusor LV10 devices (Baxter Laboratories), using 50 mL luer-lock syringes (Terumo Corporation, Tokyo, Japan). Reference standards included ceftazidime EDQM CRS (85.2% wt/wt, Sigma-Aldrich Inc, lot 4.0), pyridine analytical standard (>99%, Sigma-Aldrich Inc., Australia, lot BCCH2955), and pyridine (99.9%, Sigma-Aldrich Inc., Australia, lot SHBJ9218). All other reagents were of analytical grade or for analysis with high-pressure liquid chromatography (HPLC) (Thermo Fisher Scientific Australia Pty Ltd, Australia).

### Conditions of experiments**:** *in vitro*

The maximum daily dose of ceftazidime is recommended as 6 g per day, so this dose was used *in vitro* (20). Three ceftazidime 2 g vials totaling 6 g were reconstituted and made up in 0.9% saline to 240 mL and added to each infuser. The infusers were incubated in triplicate at 3 different temperatures: 4°C, 25°C, or 33°C for 24 h. The infuser flowed into a waste bottle, starting 30 min prior to baseline time 0 h (T = 0) to prime the line. Sample aliquots of 1 mL were taken at baseline and 4, 10, 16, 20, and 24 h of incubation and immediately frozen at –80°C. Infusers were weighed at T = 0 and 24 h to calculate the volume infused (%). Depletion of ceftazidime from infusers was measured at each temperature.

### In situ

**Fig 1:**
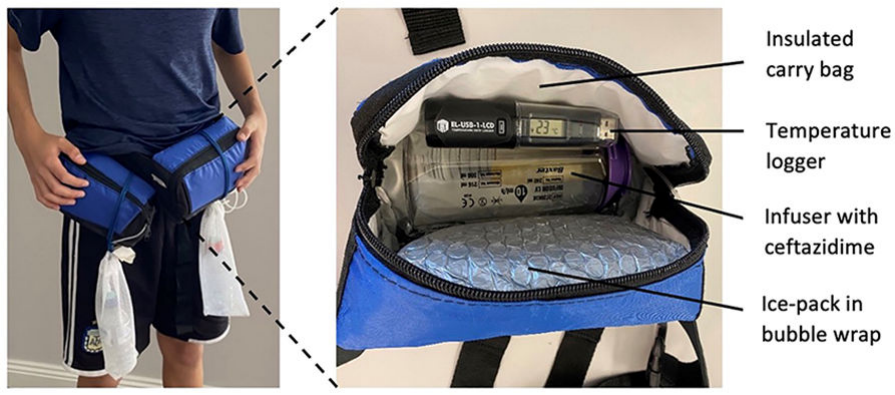
Experiment carrying two infuser bags with bottles for discard, and layout of infusion carried in an insulated bag containing one infuser, ice pack, and temperature logger.

After identifying a suitable target temperature *in vitro*, we tested whether addition of an ice pack could approximate this temperature in practice. Small Protecta chill gel ice packs (300 g) filled with nontoxic water-based refrigerant and bubble-backed were frozen at −4°C and used. Temperature was monitored over 24 h inside insulated carry bags, with and without ice packs, to confirm their effect. The impact of cooling under simulated HITH conditions was assessed with three dosing regimens: 2 g (dose for young children), 6 g (standard dose), and 12 g (higher dose in cystic fibrosis). Ceftazidime infusers were placed in insulated carry bags worn by a child volunteer for 24 h, mimicking patient use. Each volunteer wore two bags concurrently, with each bag containing an infuser at the same dose and a thermometer to log temperatures, one bag having an additional insulating ice pack wrapped in bubble wrap, and the other without (Fig. 1). Thermometers were positioned away from the body and ice pack, and infusers were delivered into waste bottles. For 12 g doses, a second ice pack was added at 12–18 h. Volunteers maintained normal daily activities, with infuser bags removed only for bathing and sleep. Samples were taken at 0, 4, 8, 12, 18, and 23 h (prior to the infusion depletion). Ceftazidime and pyridine concentrations were analyzed via HPLC. Depletion of ceftazidime from infusers was measured at each temperature, with the flow regulator taped to the skin.

### HPLC analyses

The HPLC protocol used was adopted from one previously published (21). Separation of ceftazidime and its degraded products, including pyridine, was made on a Poroshell 120, EC-C18 column (100 × 3.0 mm inner diameter; 2.7 µm; Agilent Technologies, Australia). Analyses were conducted on an Agilent Series 1100 system (Agilent Technologies, Melbourne, Australia) with a diode array UV detector. An injection volume of 5 µL and 0.4 mL/min flow with a gradient elution was used. Mobile phases consisted of ammonium formate buffer (20 mM, pH 4.5) and acetonitrile, with ratios 98:2 (vol/vol) and 85:15 (vol/vol), respectively. Under ambient temperatures, the retention time of pyridine and ceftazidime was approximately 2.5 and 10.5 min, and peaks were measured at 257 and 285 nm, respectively.

### Validation parameters

The HPLC method was validated for linearity, precision, accuracy, and detection/quantification limits. Calibration curves were generated using six freshly prepared standards: 4–400 µg/mL for ceftazidime and 0.15–100 µg/mL for pyridine, with linearity assessed over 6 days. Linearity was R^2^ 0.999 for both ceftazidime and pyridine. Intra- and inter-day precision (over 5 days) was evaluated via triplicate analysis at low, medium, and high quality control levels for ceftazidime (100, 200, and 400 µg/mL) and pyridine (0.2, 2.0, and 20 µg/mL), respectively, and expressed as relative standard deviation (%RSD). Repeatability (%) was <4.0 and <6.6, and intermediate precision (%) was <3.1 and <4.7. Accuracy was determined by spiking reference standards at three concentrations and calculating mean recovery: (measured/expected) × 100. Accuracy ranged from 94.8% to 100.4% for ceftazidime and 92.3% to 99.8% for pyridine. Limits of detection (LOD) and quantification (LOQ) were calculated per ICH Q2(R1) as LOD = 3σ/S and LOQ = 10σ/S. LOD and LOQ for pyridine were 0.07 µg and 0.22 µg, respectively.

### Sample analysis

Each sample was thawed in a 22°C water bath for 4 min and inverted 5 times before suitable dilution with mobile phase A based on the expected ceftazidime concentration. Concentrations were determined by comparing the peak area of analytes using the external-standard method of calibration. Each sample was analyzed in duplicate and the mean concentration used.

### Data analysis

Ceftazidime stability was reported as the proportion (%) of the initial ceftazidime concentration at baseline (T = 0) for comparison of ceftazidime degradation across conditions. A one-way ANOVA was performed, evaluating the effect of temperature on ceftazidime concentration, pyridine concentration, and pH. Independent *t*-tests and ANOVA were used to assess the effects of ice packs at time points; separate analyses were performed for each ceftazidime dose.

The total delivered amount of pyridine was calculated using the equation:


$$Total\;Exposure=\sum_{i=1}^{n}\frac{1}{2}\times (C_{i}+C_{i+1})\times (t_{i+1}-t_{i}).$$

Statistical analyses were performed using STATA 18 SE statistical software, with *P* < 0.05 considered significant.

## RESULTS

### *In vitro* stability

**Fig 2:**
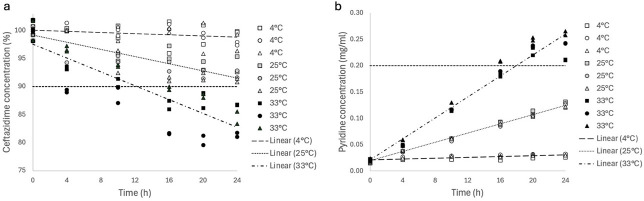
Degradation of ceftazidime 6 g incubated at 4°C, 25°C, and 33°C for 24 h: (**a**) ceftazidime concentration as the proportion of concentration at T = 0 and (**b**) pyridine concentration. Horizontal dashed line: (**a**) minimum recommended therapeutic limit of 90% and (**b**) maximum infusion concentration limit of 0.2 mg/mL.

**Fig 3:**
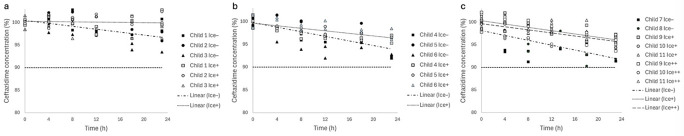
Degradation of ceftazidime in infusers carried for 24 h with no (Ice–), one (Ice+), or two (Ice++) ice packs, for (**a**) 2 g, (**b**) 6 g, and (**c**) 12 g. Horizontal dashed lines: minimum recommended therapeutic limit of 90%.

The concentration of ceftazidime in 6 g infusers compared to T = 0 decreased over time at all temperatures, but the extent was greater with higher temperatures. The concentration at 24 h remained above the predetermined 90% for infusers at 4°C and 25°C, but decreased to 84% at 33°C (Fig. 2; Table S1). Pyridine production increased with time and temperature. Starting at a concentration of 0.02 mg/mL at T = 0, by 24 h, the pyridine concentration increased at 4°C to 0.03 ± 0.002 mg/mL (increase by 50%), at 25°C to 0.13 ± 0.004 mg/mL (550%), and at 33°C to 0.24 ± 0.023 mg/mL (1,100%), in a linear manner (Fig. 3; Table S1).

The infusion flow rate decreased significantly as the temperature reduced: at 33°C, 100% of the volume was infused, at 25°C, 88% of the volume was infused, and at 4°C, only 45% of the volume was infused (*P* < 0.0001) (Table S2). Because the infuser at 33°C had depleted by 24 h and manual extraction of the 24-h sample from the outlet tubing was necessary, subsequent experiments were run over 23 h (as in previous studies). The pH of ceftazidime solutions was not affected by the incubation temperature or time (Table S3), and this was confirmed *in situ* (Table S4).

Based on the *in vitro* results, a target temperature of 25°C was deemed suitable when considering both stability and flow rate, as 33°C was unstable and the infuser flow rate at 4°C was too slow. This was, therefore, the upper temperature target for the *in situ* experiments, aiming to determine whether this could be achieved in a simple, resource-sensitive way with insulated ice packs, and measuring the resulting ceftazidime and pyridine concentrations.

### *In situ* stability in carried infusers

The age of the children wearing the carried infuser bags ranged from 11 to 16 years: 3 children carried 2 g infusers, 3 carried 6 g infusers, and 5 carried 12 g infusers. First, the effect of adding insulated ice packs on temperature was determined. Starting temperatures for the carry bags were 22°C–24.5°C. The minimum temperatures recorded for the infusers without an ice pack (Ice–), with 1 ice pack (Ice+) and with 2 sequential ice packs (Ice++), were 17.7°C, 16.6°C, and 15.3°C, respectively (Table 1). When compared to Ice–, adding ice packs significantly lowered the mean and maximum temperatures in Ice+ by 3.4°C–3.8°C (*P* < 0.001) and in Ice++ by 5.0°C–6.0°C (*P* < 0.001).

**TABLE 1:** Temperature (°C) of infusers carried over 24 h with and without ice packs

	No ice pack: Ice– Mean °C (SD) *n* = 8	1 ice pack: Ice+ Mean °C (SD) *n* = 9	2 ice packs: Ice++ Mean °C (SD) *n* = 3
Minimum	17.7 (2.3)	16.6 (1.6)	15.3 (1.5)
Mean	22.7 (1.4)	19.3 (1.5) ^*a*^	17.7 (0.6) ^*a*^
Maximum	29.2 (0.6)	25.4 (1.8) ^*a*^	23.2 (0.6) ^*a*^^,^^*b*^

^*a*^ *P *< 0.001 compared to Ice–.

^*b*^ *P *< 0.05 compared to Ice+.

The ceftazidime concentration decreased slightly over time, but for all doses (2 g, 6 g, and 12 g) and cooling conditions (Ice–, Ice+, and Ice++), its concentration remained above 90% by 23 h (Fig. 3; Table S5). All simulated infusers with and without ice packs emptied within 24 h with the flow regulator taped to the skin, showing that cooling the infuser does not impact the flow as long as the temperature of the flow regulator is impacted by being adjacent to the skin.

**Fig 4:**
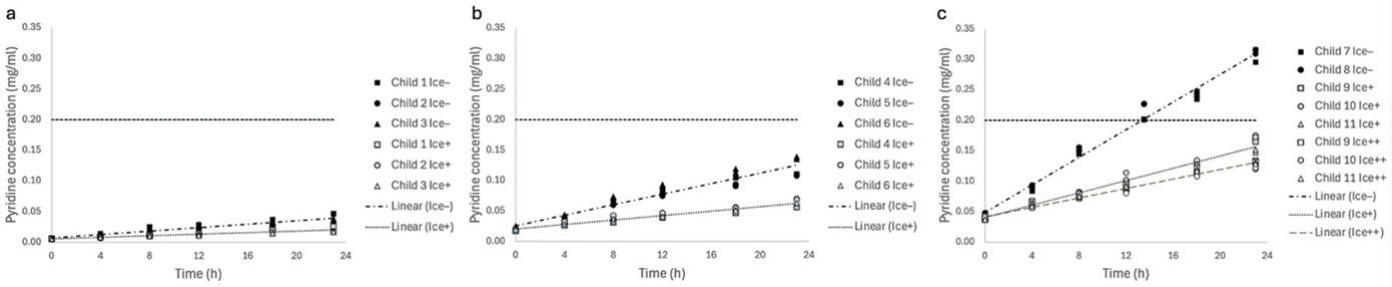
Pyridine concentration with degradation of ceftazidime in infusers carried for 24 h with no (Ice–), one (Ice+), or two (Ice++) ice packs, for (**a**) 2 g, (**b**) 6 g, and (**c**) 12 g. Horizontal dashed line: maximum recommended infusion concentration limit of 0.2 mg/mL.

The pyridine concentration with ceftazidime degradation showed a linear increase with time for ceftazidime 2 g, 6 g, and 12 g infusions (Fig. 4). Differences in the pyridine concentration between Ice– and Ice+ were apparent by 4 h, and from 8 h onward, Ice+ infusers had approximately half the pyridine concentration of Ice– (Table S6). The total delivered amount of pyridine with ceftazidime infusers was higher with higher doses and was reduced by cooling (Table 2). For each ceftazidime dose, Ice+ resulted in almost 50% reduction compared to Ice– in total pyridine production over the whole period: for 2 g pyridine, it reduced from 4.5 mg to 2.5 mg; for 6 g, it reduced from 14.7 mg to 8.0 mg. Even with the highest ceftazidime dose of 12 g, Ice+ reduced the total daily exposure from 39.1 mg to 18.8 mg, and Ice++ reduced it even slightly further to 16.7 mg.

**TABLE 2:** Total pyridine quantity delivered during the 23-h ceftazidime infusion

Exposure period	Ceftazidime 2 g pyridine quantity (mg)		Ceftazidime 6 g pyridine quantity (mg)		Ceftazidime 12 g pyridine quantity (mg)		
	Ice–	Ice+	Ice–	Ice+	Ice–	Ice+	Ice++
T = 0–4	0.3	0.3	1.1	0.9	2.5	1.9	1.7
T = 4–8	0.6	0.3	1.9	1.2	4.3	2.6	2.4
T = 8–12	0.8	0.4	2.7	1.4	6.5	3.2	2.9
T = 12–18	1.4	0.7	4.4	2.2	10.5	5.2	4.8
T = 18–23	1.4	0.8	4.5	2.3	11.4	6.0	4.9
**Total (mg)**	**4.5**	**2.5**	**14.6**	**8.0**	**39.2**	**18.8**	**16.7**

## DISCUSSION

The findings of this study show the first real-world intervention to reduce ceftazidime degradation and pyridine production to safe levels in a ceftazidime 24-h continuous infusion. The fact that the intervention is as simple and inexpensive as adding a single bubble-wrapped ice pack to an insulated bag makes it even more appealing.

The *in vitro* stage of the study aimed to determine a suitable target temperature for the *in situ* stage and also to validate our measurements against previous laboratory-based studies. Ceftazidime stability, as a proportion of the initial concentration, has been studied *in vitro* at a variety of temperatures mimicking human-body adjacent (30°C–37°C), ambient (20°C–25°C), and refrigerated (4°C). For ceftazidime at 6 or 12 g dose (in a 12% solution), at 30°C, the concentration is only 85% of initial by 24 h, and at 37°C, it only takes 8 h to reduce below 90% (4, 22). Stability can be improved by using 0.9% saline instead of 5% dextrose as a diluent, different infuser types, reduced ceftazidime concentration, and lower temperatures (14, 19). For the majority of studies, at 20°C–25°C even for a 12% solution (such as 12 g in 100mL infuser), stability remained above 90%–95% at 24 h (4, 17, 22), although in one study at 22°C, the stability dropped to 86% after 24 h (15). At 4°C, ceftazidime remains above 90% regardless of the concentration (15, 22). Our study confirms and is validated by these findings: ceftazidime is stable for 24 h at 4°C and 25°C, but drops below the therapeutic concentration of 90% after 16 h at 33°C. We chose the upper end of the 20°C–25°C ambient temperatures used in previous studies so that we could determine a safe potential maximum temperature.

The effect of temperature and time on ceftazidime degradation was also clearly shown through measurement of pyridine concentration. In one study of ceftazidime in 12% solution at 37°C, the pyridine concentration by 24 h was 4 mg/mL, but at 25°C, it reduced to 1.5 mg/mL (22). Lower concentrations of ceftazidime have lower pyridine production, but the same temperature effect (15, 22). For example, in a study by Bourget et al., for 12 g of ceftazidime in 240 mL (5% solution), the pyridine concentration at 24 h was 0.1 mg/mL at 4 °C, 0.4 mg/mL at 22 °C, and 0.8 mg/mL at 33 °C (15). This is consistent with ceftazidime (2.5% solution) in our study having pyridine concentrations at 24 h of 0.03 mg/mL at 4°C, 0.13 mg/mL at 25°C, and 0.24 mg/mL at 33°C. As others have found, flow from the infuser was reduced at 4°C, compromising the amount of ceftazidime that the patient would receive (16). Therefore, the target infuser temperature was determined to be ambient temperature, not exceeding 25°C.

For the *in situ* stage, the carried bags with ice packs stayed under this temperature limit, compared to the bags without ice packs. While the difference appears modest, this was sufficient to significantly improve ceftazidime stability and reduce pyridine production. The majority of conditions had a pyridine concentration below the EMA/USP upper limit for pharmaceutical products of 0.2 mg/mL, except the Ice–ceftazidime at 12 g (18). However, a potentially more useful metric is total pyridine exposure. There is no clear safe level for pyridine exposure, but EMA-/USP-imposed limits of environmental exposure of 2 mg per day are inappropriate for medication exposure as the former relates to regular daily exposure (18, 23, 24). Ceftazidime is not used every day, but even at the moment of reconstitution, pyridine is measurable, as shown in our study (25). There is, thus, no way of removing it completely, just reducing it to the minimum possible, especially for frequent recipients such as patients with cystic fibrosis. A previous *in vitro* study showed ceftazidime 6 g incubated at 37°C had a pyridine concentration of 0.9 mg/mL at 24 h, with the total production being 251 mg by 12 h (14). In the study by Bourget et al. with 12 g of ceftazidime, the total production of pyridine over 23 h was 16 mg at 4°C, 58 mg at 25°C, and 92 mg at 33°C (15). Their solution was to halve the dose and give 2 infusions over 12 h each, which would lead to a total delivered amount of 26 mg over 23 h at a consistent 25°C. This compares with our finding using 1 ice pack of 18.8 mg and 2 sequential ice packs of 16.7 mg, a 57% reduction compared to no ice pack. In other words, 24-h infusion with insulated ice packs results in 36% less pyridine exposure than previously reported in twice-daily 12-h infusions at 25°C. The 300 g extra weight from the ice pack was not commented on by the children in the study. The benefits of reduced pyridine and being able to receive a continuous ceftazidime infusion on HITH far outweigh this minor inconvenience.

This study solves the instability and toxicity problems previously raised. Our results show that pyridine production in 24-h ceftazidime infusions from 2 g to 12 g can be safely and effectively halved, without impacting the function of the infuser. This means the exposure is halved and patients no longer require two infusions but instead can receive their treatment once daily as a 24-h infusion for any given dose. The method is simple and inexpensive, and this is the first study to show that it works pragmatically *in situ,* as it would for patients receiving OPAT. This provides the evidence to reassure that ceftazidime can be used in a 24-h infusion, getting patients out of hospital for improved outcomes.

These findings can immediately change clinical practice. Ceftazidime continuous infusions can be used in all contexts using this solution, and most importantly in the HITH context of treatment at home, for patients with cystic fibrosis, who otherwise would have to have broader-spectrum antibiotics or stay in hospital.

## SUPPLEMENTAL MATERIAL

Supplemental material aac.01385-25-s0001.docx Tables S1 to S6.

ASM does not own the copyrights to Supplemental Material that may be linked to, or accessed through, an article. The authors have granted ASM a non-exclusive, world-wide license to publish the Supplemental Material files. Please contact the corresponding author directly for reuse.
