# Supplementary material for: Solving the degradation problem of continuous ceftazidime infusions—the Cooltaz study
Source: Antimicrob Agents Chemother. 2026 May 29;70(7):e01385-25. doi: 10.1128/aac.01385-25 (PMC13321840; doi:10.1128/aac.01385-25)
Supplement: Supplemental material — Tables S1 to S6. [file aac.01385-25-s0001.docx]

**Solving the Degradation Problem of Continuous Ceftazidime Infusions - the Cooltaz Study.**

**Supplementary data**

*In vitro experiments*

Table S1. Degradation of ceftazidime 6g incubated at 4ºC, 25ºC and 33ºC over 24h in vitro: ceftazidime concentration as proportion of concentration at T=0, and pyridine concentration

| **Time (h)** | **Ceftazidime stability**  Proportion of concentration at T=0  % (SD) | | | **­Pyridine concentration**  mg/ml (SD) | | |
| --- | --- | --- | --- | --- | --- | --- |
|  | **4°C** | **25°C** | **33°C** | **4°C** | **25°C** | **33°C** |
| T = 0 | 100.0 (1.2) | 100.0 (0.7) | 100.0 (1.6) | 0.019 (0.003) | 0.020 (0.003) | 0.021 (0.002) |
| T = 4 | 99.6 (1.6) | 98.0 (3.3) | 93.1 (3.4)* | 0.024 (0.002) | 0.039 (0.004)^†^ | 0.052 (0.005)^†‡^ |
| T = 10 | 99.6 (1.1) | 94.9 (1.4)^†^ | 91.0 (2.6)^†‡^ | 0.026 (0.003) | 0.060 (0.002)^†^ | 0.120 (0.007)^†‡^ |
| T = 16 | 99.8 (2.0) | 93.7 (2.3)^†^ | 86.0 (3.7)^†‡^ | 0.027 (0.004) | 0.089 (0.003)^†^ | 0.193 (0.013)^†‡^ |
| T = 20 | 99.0 (2.1) | 92.6 (1.2)^†^ | 85.4 (4.0)^†‡^ | 0.029 (0.003) | 0.107 (0.004)^†^ | 0.238 (0.012)^†‡^ |
| T = 24 | 98.4 (1.4) | 92.4 (1.6)^†^ | 84.2 (2.5)^†‡^ | 0.030 (0.002) | 0.126 (0.004)^†^ | 0.238 (0.023)^†‡^ |

SD: standard deviation, T = time in hours post baseline. Mean (SD) of duplicate measurements of triplicate infusers at each temperature. Ceftazidime concentration expressed as % of initial concentration (6 g/240mL) at T=0. * p<0.05, †<0.001 compared to 4ºC, ‡ p<0.001 compared to 25ºC.

Table S2. Infuser mass over 24h incubated at different temperatures in vitro.

|  | **4°C**  Mass, grams (SD) | **25°C**  Mass, grams (SD) | **33°C**  Mass, grams (SD) |
| --- | --- | --- | --- |
| **Mean T = 0** | 303.3 (2.7) | 304.2 (4.4) | 302.0 (4.7) |
| **Mean T = 24** | 199.1 (3.4) | 96.9 (4.7) | 54.1 (5.5) |
| **Mean loss** | 104.1 (2.6) | 207.3 (2.2) | 248.0 (0.8) |

T = time in hours post baseline. Mean (SD) of triplicate infusers with duplicate measurements.

Table S3. Infusion solution pH over 24h incubated at different temperatures in vitro.

| **Time (h)** | **4°C**  Mean pH (SD) | **25°C**  Mean pH (SD) | **33°C**  Mean pH (SD) |
| --- | --- | --- | --- |
| **T = 0** | 6.7 (0.1) | 6.8 (0.06) | 6.8 (0.2) |
| **T = 4** | 7.0 (0.3) | 6.8 (0.1) | 6.7 (0.3) |
| **T = 10** | 6.8 (0.3) | 6.9 (0.2) | 7.0 (0.2) |
| **T = 16** | 7.0 (0.2) | 6.8 (0.3) | 6.9 (0.4) |
| **T = 20** | 6.8 (0.2) | 7.1 (0.2) | 7.0 (0.3) |
| **T = 24** | 7.2 (0.3) | 7.0 (0.3) | 7.6 * |

T = time in hours post baseline. Mean (SD) of triplicate infusers with duplicate measurements. * Unable to measure 2 infusers due to low sample volume

*In situ* carried infuser bag experiments

Table S4. Infusion solution pH over 24h under different carried bag cooling conditions in situ.

| **Time (h)** | **Ceftazidime 2g**  Mean pH (SD) | | **Ceftazidime 6g**  Mean pH (SD) | | **Ceftazidime 12g**  Mean pH (SD) | | |
| --- | --- | --- | --- | --- | --- | --- | --- |
|  | Ice– | Ice+ | Ice– | Ice+ | Ice– | Ice+ | Ice++ |
| **T = 0** | 6.7 (0.2) | 6.5 ( 0.2) | 6.9 ( 0.1) | 6.8 ( 0.0) | 6.7 ( 0.1) | 6.8 ( 0.1) | 6.8 ( 0.1) |
| **T = 4** | 6.8 (0.1) | 6.6 ( 0.1) | 7.1 ( 0.1) | 6.9 ( 0.2) | 6.8 ( 0.1) | 7.1 ( 0.2) | 6.8 ( 0.1) |
| **T = 8** | 6.8 (0.4) | 6.6 ( 0.4) | 6.9 ( 0.1) | 6.8 ( 0.2) | 7.0 ( 0.4) | 7.1 ( 0.0) | 7.0 ( 0.2) |
| **T = 12** | 6.8 (0.1) | 6.4 ( 0.1) | 7.0 ( 0.2) | 7.2 ( 0.2) | 7.1 ( 0.0)* | 7.1 ( 0.1) | 7.0 ( 0.3) |
| **T = 18** | 6.9 (0.1) | 6.7 ( 0.1) | 7.2 ( 0.1) | 7.0 ( 0.1) | 7.0 ( 0.1) | 7.2 ( 0.1) | 7.2 ( 0.2) |
| **T = 23** | 6.8 (0.2) | 7.1 ( 0.2) | 7.2 ( 0.2) | 7.2 ( 0.1) | 7.2 ( 0.2) | 7.3 ( 0.2) | 7.1 ( 0.2) |

T = time in hours post baseline. Mean (SD) of triplicate infusers with duplicate measurements. Ice– = no ice-pack, Ice+ = one ice-pack used, Ice++ = two sequential ice-packs. * Samples taken at T=13.5h.

Table S5. Degradation of ceftazidime in infusers 2g, 6g and 12g carried by children in insulated bags with and without ice-packs over 24h. Ceftazidime concentration as proportion of concentration at T=0.

| **Time (h)** | **Ceftazidime 2g**  Mean % (SD) | | **Ceftazidime 6g**  Mean % (SD) | | **Ceftazidime 12g**  Mean % (SD) | | |
| --- | --- | --- | --- | --- | --- | --- | --- |
|  | Ice– | Ice+ | Ice– | Ice+ | Ice– | Ice+ | Ice++ |
| **T = 0** | 100.0 (1.0) | 100.0 (1.1) | 100.0 (0.8) | 100.0 (1.3) | 100.0 (1.8) | 100.0 (0.7) | 100.0 (1.9) |
| **T = 4** | 100.0 (1.6) | 100.9 (1.2) | 99.0 (2.2) | 99.2 (1.1) | 96.7 (3.8) | 99.9 (2.0) | 98.6 (1.8) |
| **T = 8** | 100.2 (2.1) | 99.8 (1.7) | 97.5 (2.4) | 97.9 (1.0) | 93.4 (1.6) | 99.1 (0.7) | 97.9 (1.5) |
| **T = 12** | 98.4 (1.5) | 99.9 (1.8) | 95.8 (2.4) | 98.0 (1.5) | 95.1(2.0)* | 97.9 (1.6) | 97.9 (1.6) |
| **T = 18** | 96.3 (1.6) | 99.9 (1.8) | 96.2 (1.8) | 97.1 (0.9) | 92.8 (2.0) | 97.0 (1.1) | 97.3 (1.8) |
| **T = 23** | 97.4 (2.5) | 100.0 (2.3) | 93.7 (2.3) | 96.7 (1.1) | 93.0 (1.6) | 96.2 (1.1) | 95.3(1.3) |

SD: standard deviation, T = time in hours post baseline. Mean (SD) of triplicate infusers with duplicate measurements. Ceftazidime concentration expressed as % of concentration (2g/240 ml, 6g/240 ml, 12g/240 ml) at T=0. Ice–: no ice-pack, Ice+: 1 ice-pack and Ice++: 2 sequential ice-packs. * Samples taken at T=13.5h.

Table S6. Pyridine concentration with degradation of ceftazidime 2g, 6g and 12g in infusers carried by children in insulated bags with and without ice-packs over 24h.

| **Time (h)** | **Ceftazidime 2g**  mg/ml (SD) | | **Ceftazidime 6g**  mg/ml (SD) | | **Ceftazidime 12g**  mg/ml (SD) | | |
| --- | --- | --- | --- | --- | --- | --- | --- |
|  | Ice– | Ice+ | Ice– | Ice+ | Ice– | Ice+ | Ice++ |
| **T = 0** | 0.006 (0.0002) | 0.006 (0.0001) | 0.020 (0.002) | 0.019 (0.001) | 0.044 (0.004) | 0.040 (0.002) | 0.039 (0.002) |
| **T = 4** | 0.011 (0.002) | 0.008 (0.001) | 0.040 (0.003) | 0.030 (0.003) | 0.089 (0.004) | 0.065 (0.002) | 0.059 (0.002) |
| **T = 8** | 0.020 (0.004) | 0.011 (0.001) | 0.066 (0.006) | 0.037 (0.004) | 0.150 (0.004) | 0.079 (0.003) | 0.074 (0.002) |
| **T = 12** | 0.027 (0.003) | 0.012 (0.001) | 0.085 (0.007) | 0.043 (0.003) | 0.214 (0.015)* | 0.098 (0.009) | 0.087 (0.004) |
| **T = 18** | 0.032 (0.004) | 0.017 (0.002) | 0.104 (0.011) | 0.052 (0.003) | 0.242 (0.006) | 0.124 (0.010) | 0.116 (0.005) |
| **T = 23** | 0.038 (0.008) | 0.020 (0.004) | 0.119 (0.014) | 0.063 (0.006) | 0.309 (0.010) | 0.164 (0.011) | 0.128 (0.005) |

T = time in hours post baseline. Pyridine concentration expressed as mean (SD) of triplicate infusers with duplicate measurements. Ice–: no ice-pack, Ice+: 1 ice-pack and Ice++: 2 sequential ice-packs. * Samples taken at T=13.5h.
